# Toward a new paradigm of care: a surgical leaders’ Delphi consensus on the organizational factors of the new pancreas units (E-AHPBA PUECOF study)

**DOI:** 10.1007/s13304-024-01839-x

**Published:** 2024-04-25

**Authors:** Lorenzo Cobianchi, Francesca Dal Mas, Mohammad Abu Hilal, Mustapha Adham, Sergio Alfieri, Gianpaolo Balzano, Giedrius Barauskas, Claudio Bassi, Marc G. Besselink, Maximilian Bockhorn, Ugo Boggi, Kevin C. Conlon, Roberto Coppola, Christos Dervenis, Safi Dokmak, Massimo Falconi, Giuseppe Kito Fusai, Andrew A. Gumbs, Arpad Ivanecz, Riccardo Memeo, Dejan Radenković, Jose M. Ramia, Elena Rangelova, Roberto Salvia, Alain Sauvanet, Alejandro Serrablo, Ajith K. Siriwardena, Stefan Stättner, Oliver Strobel, Alessandro Zerbi, Giuseppe Malleo, Giovanni Butturini, Isabella Frigerio

**Affiliations:** 1 Department of Clinical, Surgical, Diagnostic and Pediatric Sciences, University of Pavia, Via Alessandro Brambilla, 74, 27100 Pavia, Italy; 2 Pancreas Unit Directorship, IRCCS Policlinico San Matteo Foundation, Pavia, Italy; 3 Collegium Medicum, University of Social Sciences, Łodz, Poland; 4 Department of Management - Venice School of Management, Ca’ Foscari University, Venice, Italy; 5 Department of General Surgery, Istituto Ospedaliero Fondazione Poliambulanza, Brescia, Italy; 6 Department of Hepato-Biliary and Pancreatic Surgery, Edouard Herriot Hospital, Hospices Civils De Lyon, Lyon, France; 7 Digestive Surgery, Fondazione Policlinico Universitario A. Gemelli, IRCCS, Catholic University, Rome, Italy; 8 Pancreatic and Transplant Surgery Unit, Pancreas Translational and Clinical Research Center, IRCCS Ospedale San Raffaele, ENETS Center of Excellence, Milan, Italy; 9 Department of Surgery, Lithuanian University of Health Sciences, Kaunas, Lithuania; 10 Department of Surgery, Amsterdam UMC, University of Amsterdam, Amsterdam, The Netherlands; 11 Cancer Center Amsterdam, Amsterdam, The Netherlands; 12 Department of General and Visceral Surgery, University Medical Centre Oldenburg, Oldenburg, Germany; 13 Division of General and Transplant Surgery, University of Pisa, Pisa, Italy; 14 Department of Surgery, School of Medicine, Trinity College, Dublin, Ireland; 15 Centre for Pancreatico-Biliary Diseases, Department of Surgery, Tallaght University Hospital, Dublin, Ireland; 16 General Surgery, Università Campus Bio-Medico di Roma, Rome, Italy; 17 General Surgery, Fondazione Policlinico Universitario Campus Bio-Medico, Rome, Italy; 18 Department of Hepatobiliary and Pancreatic Surgery, Metropolitan Hospital, Piraeus, Greece; 19 Department of Hepatobiliopancreatic Surgery and Liver Transplantation, Beaujon Hospital, GHU AP-HP.Nord-Université Paris Cité, Paris, France; 20 Department of HPB Surgery and Liver Transplant, Royal Free Hospital NHS Foundation Trust, London, UK; 21 Division of Surgery and Interventional Sciences, University College London, London, UK; 22 Advanced & Minimally Invasive Surgery Excellence Center, Department of Surgery, American Hospital Tbilisi, Tbilisi, Georgia; 23 Department of Abdominal and General Surgery, University Medical Center Maribor, Maribor, Slovenia; 24 Unit of Hepato-Pancreato-Biliary Surgery, General Regional Hospital “F. Miulli”, Acquaviva Delle Fonti, Italy; 25 Faculty of Medicine, University of Belgrade, Belgrade, Serbia; 26 Clinical Center of Serbia, Clinic of Digestive Surgery, Belgrade, Serbia; 27 Department of Surgery, Hospital General Universitario Dr. Balmis, Alicante, Spain; 28 Division of Surgery, Department of Clinical Science, Intervention, and Technology (CLINTEC), Karolinska Institutet, Stockholm, Sweden; 29 Section for Upper Abdominal Surgery at Department of Surgery, Sahlgrenska University Hospital, Gothenburg, Sweden; 30 Department of Surgery, Dentistry, Pediatrics and Gynecology, University of Verona, Verona, Italy; 31 General and Pancreatic Surgery Department, Pancreas Institute, University of Verona Hospital Trust, Verona, Italy; 32 Université Paris Cité, Centre de Recherche Sur l’InflammationInserm, Paris, France; 33 Department of Hepatobiliary Surgery, APHP Nord Beaujon Hospital, Clichy, France; 34 Department of Surgery, Miguel Servet University Hospital, Zaragoza, Spain; 35 Hepato-Pancreato-Biliary Unit, Manchester Royal Infirmary, Manchester, UK; 36 Department of General, Visceral and Vascular Surgery, Centre for Hepatobiliary Surgery, Vöcklabruck, Austria; 37 Medizinische Universitat Wien, Vienna, Austria; 38 Department of Biomedical Sciences, Humanitas University, Pieve Emanuele, Italy; 39 Pancreatic Surgery Unit, Humanitas Research Hospital -IRCCS, Rozzano, Italy; 40 Department of HPB Surgery, Pederzoli Hospital, Peschiera del Garda, Italy; 41 Verona, Italy

**Keywords:** Pancreatic cancer, Pancreas units, Disease units, Organizational model, Delphi

## Abstract

Pancreas units represent new organizational models of care that are now at the center of the European debate. The PUECOF study, endorsed by the European–African Hepato-Pancreato-Biliary Association (E-AHPBA), aims to reach an expert consensus by enquiring surgical leaders about the Pancreas Units’ most relevant organizational factors, with 30 surgical leaders from 14 countries participating in the Delphi survey. Results underline that surgeons believe in the need to organize multidisciplinary meetings, nurture team leadership, and create metrics. Clinical professionals and patients are considered the most relevant stakeholders, while the debate is open when considering different subjects like industry leaders and patient associations. Non-technical skills such as ethics, teamwork, professionalism, and leadership are highly considered, with mentoring, clinical cases, and training as the most appreciated facilitating factors. Surgeons show trust in functional leaders, key performance indicators, and the facilitating role played by nurse navigators and case managers. Pancreas units have a high potential to improve patients' outcomes. While the pancreas unit model of care will not change the technical content of pancreatic surgery, it may bring surgeons several benefits, including more cases, professional development, easier coordination, less stress, and opportunities to create fruitful connections with research institutions and industry leaders.

## Introduction

Pancreatic cancer (PC) has the worst prognosis among solid malignancies and carries the most remarkable mortality rate. Although clinical research and practice have increased the likelihood of prolonged survival in recent years, the diagnostic and therapeutic path remains extremely demanding. When feasible, radical surgery combined with systemic chemotherapy offers the highest chances for long-term survival, with highly specialized centers in pancreatic surgery leading to better clinical outcomes when compared to low-volume ones [1–5]. The majority of patients diagnosed with PC will need palliative and end-of-life care [6–8].

Each phase of the clinical pathway, from first diagnosis to therapeutic decisions and follow-up, requires specialized knowledge and skills. Among these, surgery represents a crucial step, together with histopathology, medical oncology, radiotherapy, and palliative care. Nonetheless, each patient must be treated for their unique nutritional [9, 10] and psychological [11, 12] needs. Integrating simultaneous and palliative treatment, from the earliest phases to end-of-life care, requires a multidisciplinary approach to a care continuum.

As translational research is often slow, improvement can be found in organizational innovation, namely, designing a different, most efficient, and effective patient’s journey. The so-called *disease units* represent new organizational, patient-centric models of care, where various medical professionals and stakeholders share their contributions and knowledge, and actions are tailored according to the disease and the single patient’s needs [13]. In general terms, a disease unit differs from a simple multidisciplinary board in several aspects, including:

- A more comprehensive number and variety of stakeholders involved in the process beyond medical professionals.
- The presence of professional figures aiming at bridging the knowledge and organizational gaps between the patient and the medical staff, fostering, whenever possible, co-production and shared decision-making dynamics [14–17].
- The creation, collection, and sharing of medical information about the patient, but also in an aggregate way for clinical and research purposes.
- The definition and monitoring of each step of care by balancing those medical activities that may be more complex or require higher specialization or instruments than those that may be easily present in less specialized centers.

The European debate on *disease units* is now lively when it comes to PC. Pancreas units, depending on national or regional regulation, are requested to have specified characteristics and minimal requirements, leaving to hub centers defined activities (i.e., surgery) and to spokes some others (i.e., endoscopy, medical treatment, biopsies). Still, there is an open debate and lack of literature on how these pancreas units should be designed, which organizational model they should follow, and how knowledge should be created, captured, and shared in a multistakeholders’ approach.

Starting from these premises, the PUECOF (Pancreas Unit Expert Consensus on the Organizational Factors) study aims to test a possible organizational model through a Delphi panel, seeking an expert consensus among surgical leaders involved in the European pancreatic surgery scenario. In their role as hybrid managers with some managerial responsibilities [18, 19], surgical leaders represent key figures in setting up the “rules of the games” in establishing such new organizational structures. The study was endorsed by the European–African Hepato-Pancreato-Biliary Association (E-AHPBA).

## Methods

### Design and setting

A Delphi survey [20, 21] was designed starting from the recent literature on healthcare organization science [13], stakeholders’ engagement [14], knowledge translation [22], and non-technical skills [23–25]. The survey was conceived by a multidisciplinary panel encompassing scholars in general surgery, healthcare management, organization science, social statistics, and healthcare policies. The final version of the survey was revised by the steering committee (LC, FDM, GM, GBu, and IF) and audited by E-AHPBA to grant the endorsement, which was given in October 2022.

The experts included in the panel were gathered from the members’ list of E-AHPBA. The criteria for selecting the participants were: renowned leadership in pancreatic surgery practice and research within the E-AHPBA community, affiliation to high-volume centers, and diversity in gender and countries to cover most of the E-AHPBA area of interest.

Thirty-eight surgical leaders from 17 countries were personally invited via email to join the initiative. The project was advertised on the E-AHPBA website, although participation was by invitation only. Thirty experts accepted the invitation and participated in the first round. Twenty-six experts participated in the second round, with a response rate of 87%. Data collection was carried out between November 2022 and March 2023 through Google Forms.

### Survey and statistical analysis

The Delphi survey included some descriptive statistics and four sections, namely the organizational factors (14 items), the stakeholders (18 items), the non-technical skills (1 + 16 items), and the processes and knowledge translation facilitators (31 items). All items needed to be voted on by the expert panel on a Likert scale from 1 (not important) to 9 (very important). A positive consensus (inclusion) was gathered for those items voted with a mean of over 7. A negative consensus (exclusion) was defined for those items voted with a mean of 3 or below.

Another round of discussion had to be made for those items voted from 3.1 to 6.9 as the average. The Delphi round 2 survey was designed to revote the factors that did not reach a consensus by expressing the same macro-concepts in a different way than round 1 to avoid bias. Fifteen items were brought forward in round 2.

The Delphi questionnaires for both round 1 and 2, and the definition of the macro-topics brought forth in round 2 are reported in Appendix 1.

Statistical analysis was conducted using R (RStudio version 2023.06.1.524) [26].

## Results

### Descriptive statistics

Thirty surgical leaders responded to round 1. The sample included practitioners from 14 countries, with Italy as the most represented one (12 participants, equal to 40%). Surgeons were mostly males, with only one female. The vast majority of them declared more than 20 years of experience in pancreatic surgery (21, 70% of the sample), and most of them were from academic institutions (26, 87%). 60% of them hold leadership roles as department chiefs, 73% of them were over 50 years old, more than half of the sample was from high-volume centers, with more than 81 surgical resections performed per year.

Table 1 reports some of the descriptive statistics about the sample.

**Table 1:** Descriptive statistics about the sample

Number of surgical leaders	30	
*Represented countries*	14	100.00%
Austria	2	6,67%
France	3	9,99%
Germany	1	3.33%
Grece	1	3.33%
Georgia	1	3.33%
Ireland	1	3.33%
Italy	12	40.00%
Lithuania	1	3.33%
Serbia	1	3.33%
Slovenia	1	3.33%
Spain	2	6.67%
Sweden	1	3.33%
The Netherlands	1	3.33%
UK	2	6.67%
*Gender*		100.00%
Male	29	96.67%
Female	1	3.33%
*Years of experience in pancreatic surgery*		100.00%
11–20 years	9	30.00%
> 20 years	21	70.00%
*Kind of institution*		100.00%
Academic	26	86.67%
Non-academic	4	13.33%
*Current position*		100.00%
Board-certified surgeon	2	6.67%
Senior consultant	10	33.33%
Head of department	18	60.00%
Age group		100.00%
41–50 years	8	26.67%
50–60 years	12	40.00%
Over 60 years	10	33.33%
*Number of yearly pancreatic resections*		100.00%
< 30 resections	1	3.33%
From 31 to 50 resections	4	13.33%
From 51 to 80 resections	8	26.67%
> 81 resections	17	56.67%

### ***Section 1: organizational factors***

The first section was about the organizational factors that were most important when establishing a pancreas unit. Among the initial 14 factors, half of them reached a consensus in round 1, while the others had to be brought forward in round 2.

The best-rated factor was the need to organize frequent meetings about multidisciplinary clinical professionals, which is an already common task in the multidisciplinary board. Other highly rated factors included the definition of team leaders for each clinical and organizational function, the presence of metrics and key performance indicators to assess the quality of the healthcare service, and the need to introduce case managers to support and guide the patient throughout the journey.

The items to be brought forth included the use of technology (e.g., telemedicine tools) to support the relationships with both staff and patients and knowledge dissemination through the press and media.

Table 2 reports the results of round 1 concerning the section about the organizational factors.

**Table 2:** Organizational factors: round 1 results

Organizational factors	Mean	SD	Round 1	Round 2 topic
Organizing frequent meetings among multidisciplinary clinical professionals	7.93	1.00		
Defining team leaders for each clinical and organizational function	7.53	1.33		
The presence of metrics/key performance indicators assessing all steps of the patient journey	7.30	1.37		
Introducing “case managers”, in charge to follow the patient and the case over different clinic departments/expertise	7.27	1.48		
Sharing knowledge, cases, … with other pancreas units	7.07	1.50		
Defining multidisciplinary clinical training paths for all the unit’s staff members	7.00	1.34		
Covering all patients journey, in an “extended view”, starting from prevention (e.g., promotion of good habits like avoiding smoke and alcohol and enhancing physical activity) and early diagnosis (increasing awareness in familial cases through National Registry inclusion; spreading the culture about the most common non-specific symptoms such as back pain, diabetes, wight loss, steatorrhea,..)	6.90	1.60		
Integrating data across clinical and administrative unit	6.83	1.39		Data integration
Defining multidisciplinary management/organizational training paths for all the unit’s staff members	6.77	1.50		Multidisciplinary education
Having spaces fully dedicated to the pancreas unit	6.63	1.80		Dedicated visual spaces
Using new technologies (e.g., telemedicine) in managing collaboration and work relations within the unit’s staff	6.40	1.69		Telemedicine among staff
Using new technologies (e.g., telemedicine) in managing the relations with the patients	6.20	1.99		Telemedicine for patients
Disseminate knowledge by being present on the local press	5.23	1.99		Using the local press
Disseminate knowledge by being present on social media	5.03	1.92		Using social media networks

In round 2, all the remaining items got full consensus. However, the sample was not equally distributed, especially about the potential contribution of press and media, which recorded a standard deviation of 1.94, dividing the participants between those more open to the new tools and those skeptical.

Table 3 reports the results of round 2 concerning the section about the organizational factors.

**Table 3:** Organizational factors: round 2 results

Organizational factors	Round 2 topic	Macro-topic for assessment	Mean	SD	Round 2
Integrating data across clinical and administrative unit	Data integration	Use of technology and telemedicine	7.29	1.61	
Defining multidisciplinary management/organizational training paths for all the unit’s staff members	Multidisciplinary education	Professional and research network	8.38	1.34	
Having spaces fully dedicated to the pancreas unit	Dedicated visual spaces	Patients' support network	7.22	1.76	
Using new technologies (e.g., telemedicine) in managing collaboration and work relations within the unit’s staff	Telemedicine among staff	Use of technology and telemedicine	7.29	1.61	
Using new technologies (e.g., telemedicine) in managing the relations with the patients	Telemedicine for patients	Use of technology and telemedicine	7.29	1.61	
Disseminate knowledge by being present on the local press	Using the local press	Press and media	7.29	1.94	
Disseminate knowledge by being present on social media	Using social media networks	Press and media	7.29	1.94	

### ***Section 2: stakeholders***

The second section was about the stakeholders to be engaged in a pancreas unit. Notably, among the initial 18 subjects, only 3 of them (17%) reached a consensus in round 1, while the remaining 15 had to be brought forward in round 2.

The only actors that were recognized as necessary were those traditional medical professionals (like surgeons, oncologists, radiation therapists, and palliative care specialists) who are part of the already-established care path. These actors received an overall rating of 8.4 out of 9 and a low standard deviation of 0.61. The other two stakeholders who received a consensus (still with lower degrees) were non-traditional clinical professionals (like physiotherapists and psychologists) and the patient.

Table 4 reports the results of round 1 concerning the section about stakeholders.

**Table 4:** Stakeholders: round 1 results

Stakeholders	Mean	SD	Round 1	Round 2 topic
Involving traditional clinical professionals (e.g., surgeons, oncologists, radiation therapists, palliative care specialists, …)	8.40	0.61		
Involving non-traditional clinical professionals (physiotherapists, psychologists, …)	7.53	1.02		
Involving the patient	7.43	1.50		
Involving clinicians from partner institutions according to a hub/spokes model	6.93	1.41		Hub and spokes relations
Involving the patient’s family	6.83	1.53		Relationships with the family
Involving clinicians from international institutions	6.80	1.70		Relationships with the international community
Involving other pancreas units	6.80	1.72		Relationships with other pancreas units
Involving pure research institutions and universities	6.70	1.64		Relationships with universities
Involving scientific societies	6.47	1.80		Relationships with scientific societies
Involving the patients’ associations	6.27	1.93		Relationships with patients associations
Involving ethical committees	5.93	2.06		Relationships with ethics committees
Involving colleagues characterized by diverse features	5.83	1.98		Diverse team members
Involving private firms/companies active in the surgical technology sector	5.53	1.77		Relationships with tech providers
Involving other public sector entities	5.50	1.96		Relationships with public sector entities
Involving private firms/companies active in the pharma sector	5.47	1.98		Relationships with pharma
Involving private firms/companies active in the biotech sector	5.47	1.98		Relationships with biotech firms
Involving other NGOs (non-governmental organizations) and no-profit entities	5.33	1.92		Relationships with NGOs
Involving private firms/companies active in the media sector	5.03	1.70		Relationships with the media

Most of the stakeholders received a consensus while reconnected to those macro-themes brought forth to round 2, except for private firms and industry leaders (mean 5.99, standard deviation 2.12). Again, a high standard deviation denotes that the sample is widely distributed.

Table 5 reports the results of round 2 concerning the section about stakeholders.

**Table 5:** Stakeholders: round 2 results

Organizational factors	Round 2 topic	Macro-topic for assessment	Mean	SD	Round 2
Involving clinicians from partner institutions according to a hub/spokes model	Hub and spoke relations	Professional and research network	8.38	1.34	
Involving the patient’s family	Relationships with the family	Patients' support network	7.22	1.76	
Involving clinicians from international institutions	Relationships with the international community	Professional and research network	8.38	1.34	
Involving other pancreas units	Relationships with other pancreas units	Professional and research network	8.38	1.34	
Involving pure research institutions and universities	Relationships with universities	Professional and research network	8.38	1.34	
Involving scientific societies	Relationships with scientific societies	Professional and research network	8.38	1.34	
Involving the patients’ associations	Relationships with patients associations	Patients' support network	7.22	1.76	
Involving ethical committees	Relationships with ethics committees	Professional and research network	8.38	1.34	
Involving colleagues characterized by diverse features	Diverse team members	Professional and research network	8.38	1.34	
Involving private firms/companies active in the surgical technology sector	Relationships with tech providers	Use of technology and telemedicine	7.29	1.61	
Involving other public sector entities	Relationships with public sector entities	Patients' support network	7.22	1.76	
Involving private firms/companies active in the pharma sector	Relationships with pharma	Industrial partners	5.99	2.12	
Involving private firms/companies active in the biotech sector	Relationships with biotech firms	Industrial partners	5.99	2.12	
Involving other NGOs (non-governmental organizations) and no-profit entities	Relationships with NGOs	Patients' support network	7.22	1.76	
Involving private firms/companies active in the media sector	Relationships with the media	Press and media	7.29	1.94	

### ***Section 3: non-technical skills***

All the items about non-technical skills receive full consensus in round 1, without discussing and evaluating the topic further. Interestingly, the worst-rated factor was the importance of non-technical or soft skills in general terms. The best-rated skills were ethics, teamwork, professionalism, and leadership.

Table 6 reports the results of the section about non-technical skills.

**Table 6:** Non-technical skills: round 1 results

Non-technical skills	Mean	SD	Round 1	Round 2 topic
Ethics	8.77	0.50		
Team working	8.70	0.53		
Professionalism	8.60	0.61		
Leadership	8.57	0.67		
Decision-making	8.53	0.85		
Communication	8.43	0.80		
Planning ability	8.20	0.75		
Learning ability	8.20	0.75		
Information management ability	8.17	0.82		
Multicultural ability	8.13	1.02		
Situational awareness	8.10	0.83		
Managing stress	8.00	0.93		
Ideas creation ability	7.80	1.40		
Agility	7.63	1.52		
Coping with fatigue	7.57	1.12		
Coordination ability	7.53	1.52		
*Non-technical or soft skills in general terms*	*7.43*	*1.31*		

### ***Section 4: facilitators***

The last section concerned the tools (technical, non-technical, technological, and creative) that could facilitate the knowledge-sharing dynamics within a pancreas unit. Notably, all the items received full consensus in round 1, without the need to continue the dialog in round 2. Still, not all the factors received the same level of agreement and importance.

The best-rated facilitators included mentoring and leadership, clinical cases, participation in national and international meetings, training, and clinical guidelines. The worst-rated items were co-production, leaflets and brochures, design elements, and testimonials.

Table 7 reports the results of the section about facilitators.

**Table 7:** Facilitators: round 1 results

Facilitators	Mean	SD	Round 1	Round 2 topic
Mentoring and leadership	8.53	0.62		
Clinical cases and critical review of complicated cases	8.37	0.84		
Participation in national and international meetings	8.37	0.75		
Training	8.27	0.81		
Clinical guidelines	8.13	1.02		
Use of evidence-based methods	8.10	1.11		
Mobile electronic medical records and online tools	8.07	0.85		
Discussions, debates, curiosity	8.00	0.93		
Lesson learned and best practices	7.97	1.02		
Use of interpersonal skills	7.90	1.08		
Quality assessment by stakeholders	7.90	0.94		
Journal publications	7.87	1.63		
Self-assessment	7.80	1.01		
In-person visit and talking	7.77	1.63		
Establishment of mixed teams	7.73	0.85		
The pathogenesis and mechanisms behind diseases	7.70	1.22		
Committees and meetings	7.70	1.10		
New technological tools	7.63	1.17		
Use of a simple language	7.63	1.43		
Empowerment	7.53	1.26		
Engaging with the patient's family	7.47	1.31		
Multidisciplinary people (e.g., degree in medicine + IT)	7.40	1.47		
Image tagging	7.33	1.32		
Simulations	7.33	1.64		
Community of practice	7.30	1.35		
Tours to share experiences with others	7.27	1.24		
Web portals	7.20	1.17		
Co-production	7.17	1.34		
Leaflets and brochures	7.13	0.99		
Design	6.93	1.09		
Testimonials	6.93	1.36		

## Discussion

The new pancreas units represent a unique and immense opportunity for pancreatic cancer care, including raising the quality of care for patients and the quality of work for clinical professionals. The new organizational model may bring several benefits to pancreatic surgeons, for instance, in the number of treated cases, professional development through networking, easier coordination with colleagues and patients thanks to the formal establishment of professional figures such as case managers, and more opportunities to connect with research institutions and industry leaders for joint projects and trials. Moreover, one more potential benefit of creating pancreas units may be reducing pancreatic surgeon burnout. Indeed, pancreatic surgery has elevated morbidity and mortality when compared to essentially every other intracavitary procedure. The development of pancreas units may enhance camaraderie among pancreatic surgeons, and the added support of non-surgical pancreatic experts such as round-the-clock interventional radiologists and endoscopists could help mitigate the difficulties of providing pancreatic surgical care [27, 28].

The debate about the most meaningful organizational factors, stakeholders, and procedures to be established in this new care concept is still open, and so is the design of an “ideal” pancreas unit. The results of the PUECOF study are intriguing and can contribute to the current debate on organizational innovation in surgery.

Regarding the organizational factors and the meaningful stakeholders, the enquired surgical leaders still seem stuck to a traditional model of care. Indeed, the best-rated item was about the need to carry on multidisciplinary meetings to discuss the patient’s case, a routine task that represents the primary paradigm in surgical oncology today. In line with this result, surgeons feel more comfortable enquiring those medical professionals who are part of the tumor board, such as surgeons, oncologists, radiation therapists, and palliative care specialists. They are also open to connecting and discussing with less traditional medical professionals, for instance, in the fields of nutrition and psychology, as recommended by the latest literature on pancreatic cancer care [9–12].

Interestingly enough, surgeons consider patients as relevant parties, while other stakeholders are not, neither the patient’s family nor the caregivers. These results are in contrast with the recognized pillars of the modern healthcare system, which is based on patient-centric concepts like co-production and shared decision-making. Modern oncology requires clinicians to engage the patient as much as possible, considering the circumstances [29–31], to co-design, co-plan, co-decide, and co-execute the oncological care [14, 32]. The concept of co-production has recently been also applied to surgery [14], and one first experience has been planned also in pancreatic cancer care [33]. Patients who are fully engaged in their own care record higher levels of satisfaction and adherence to the care plan [29, 30]. Therefore, it is important even for surgeons to second these elements when they are feasible.

Surgeons are also less likely to engage with other stakeholders, such as other clinicians from partner institutions or other pancreas units, universities, scientific societies, patients’ associations, NGOs, and industry firms. Surgical leaders seem less willing to go far beyond their operating room, as the meaningful partners they need are just outside it. Also these findings are in contrast with the most recent trends describing the healthcare ecosystem as an open one [34, 35], and the need for a multistakeholder approach [14], in which each actor has a role and can contribute to the ultimate goal, namely, the patient’s satisfaction and quality of care.

However, the role of professional figures such as nurse navigators, case managers [36], or advanced nurse practitioners [37] emerges. These professionals aim to connect the patients and the clinical staff, offering technical and non-technical support throughout the entire care journey. Surgeons are also sensitive to the organizational dimension through the definition of functional leaders and chiefs and the establishment of metrics and key performance indicators. In contrast, there is less trust in the use of telemedicine to support the relationships between medical professionals and the patient.

About the non-technical skills, in line with the current surgical literature [23, 25], surgical leaders recognized their importance. The best-rated items are represented by ethics, teamwork, professionalism, leadership, decision-making, and communication, which stress the importance of team dynamics within the operating room [38]. Interestingly, less importance is devoted to managing stress, ideas creation ability, agility, coping with fatigue, and coordination, even if pancreatic surgery is known to be technically complex with long operating times [39, 40].

Last but not least, concerning the facilitators, again, surgeons seem more comfortable when employing traditional instruments, like mentoring, clinical reports and critical review of complicated cases, participation in national and international meetings, training, clinical guidelines, and evidence-based methods. They have less trust in modern concepts like the above-mentioned co-production or marketing-oriented tools like leaflets and brochures, design and visual elements, and testimonials, who may share their personal experience with others.

Although the establishment of pancreas units will not change the technical content of pancreatic surgery, it can foster the progress of research and innovation thanks to the multistakeholder perspective in terms of new pharmaceutical treatments to improve resection rates, surgical techniques, or new frontiers in the use of telemedicine and e-health tools in pancreatic care and follow-up. It is an exciting scenario where everyone must contribute and offer an effort to change the paradigm to fight one of the worst oncological diseases.

## Conclusions

The debate on the new pancreas units has just started. Our exciting results depict a picture that sees surgical leaders still comfortable in their traditional role. Indeed, those organizational factors that reached full consensus are those closer to the actual situation and procedures, while surgeons are still reluctant to recognize more modern concepts like multistakeholder engagement and co-production dynamics.

The above-described scenario has a substantial impact on new essential needs in surgical education for the next generation of surgical oncologists. Both academic institutions and scientific societies will have the strategic role of promoting such a paradigm shift, sharing knowledge and best practices about what is and will go on in pancreatic cancer care.

### Limitations

As with all pieces of research, ours has limitations. First of all, our Delphi panel members are not equally distributed, with many coming from Italy, where the debate on pancreas units is particularly lively because of the recent regulation of the Lombardy Region [13, 41] and the creation of a dedicated focus group by the Ministery of Health [42]. Despite our efforts, gender diversity is not met, with only 1 woman out of 30 total participants, reflecting the scenario of a (still) male-dominated domain. Moreover, the literature and the topic of pancreas units are still in their infancy. As the theme progresses, we may expect more intriguing inputs and experiences to be included in the debate. Inquiring different clinical professionals other than surgeons, such as oncologists, gastroenterologists, and also nurses, about their perception of the meaningful organizational factors may create valuable synergies with the thoughts of surgical leaders. Our limitations can, therefore, represent new exciting research avenues for researchers in surgery and healthcare management to design, disseminate, and assess the “ideal” pancreas unit model of care.

## Data Availability

The datasets generated during and/or analyzed during the current study are available from the corresponding author upon reasonable request.

## Appendix

### Delphi questionnaire: round 1

HPB surgical leaders’ expert consensus on the organizational factors of a formally established pancreas unit

#### Section 0: descriptive statistics

1. How many years of experience in pancreatic surgery do you have?

1.  < 10
2. 11 to 20
3.  > 20

2. What kind of institution do you work for?

1. Academic
2. Non-academic

3. What is your current position?

1. Board-certified surgeon
2. Senior consultant
3. Head of the department

4. How many pancreatic resections does your institution perform every year?

1.  < 30
2. from 31 to 50
3. from 51 to 80
4. over 81

5. What is your gender?

1. Male
2. Female
3. Prefer not to answer

6. What is your age group?

1. Under 40
2. 1–50
3. 50–60
4. Over 60

7. Do you agree to be an author in the scientific publication(s) reporting the Delphi results?

1. Yes

#### Section 1: organizational factors (14 items)

The aim of the section is to gather expert consensus on the importance of some organizational factors. Although all of them may seem perceived as relevant, please assess them by ranking seven and up only those elements that, to you, should have the priority in the establishment of a multidisciplinary pancreas unit.

**Table:**

In a pancreas unit, how would you rate the importance of the following items from 1 to 9, where: 1 = not important at all, 9 = very important, when it comes to the organizational factors in pancreatic cancer care?		Importance								
		1	2	3	4	5	6	7	8	9
1	Organizing frequent meetings among multidisciplinary clinical professionals									
2	The presence of metrics/key performance indicators assessing all steps of the patient journey									
3	Having spaces fully dedicated to the pancreas unit									
4	Defining team leaders for each clinical and organizational function									
5	Using new technologies (e.g., telemedicine) in managing collaboration and work relations within the unit’s staff									
6	Using new technologies (e.g., telemedicine) in managing the relations with the patients									
7	Sharing knowledge, cases, … with other pancreas units									
8	Disseminate knowledge by being present on the local press									
9	Disseminate knowledge by being present on social media									
10	Defining multidisciplinary clinical training paths for all the unit’s staff members									
11	Defining multidisciplinary management/organizational training paths for all the unit’s staff members									
12	Integrating data across clinical and administrative unit									
13	Introducing “case managers”, in charge to follow the patient and the case over different clinic departments/expertise									
14	Covering all patients journey, in an “extended view”, starting from prevention (e.g., promotion of good habits like avoiding smoke and alcohol and enhancing physical activity) and early diagnosis (increasing awareness in familial cases through National Registry inclusion; spreading the culture about the most common non-specific symptoms like back pain, diabetes, wight loss, steatorrhea,..)									

#### Section 2: stakeholders (18 items)

The aim of the section is to gather expert consensus on the importance of engaging certain stakeholders, than others. Although all of them may seem perceived as relevant, please assess them by ranking seven and up only those actors that, to you, should have the priority in the establishment of a multidisciplinary pancreas unit.

**Table:**

In a pancreas unit, how would you rate the importance of the following items from 1 to 9, where: 1 = not important at all 9 = very important when it comes to stakeholders' engagement in pancreatic cancer care?		Importance								
		1	2	3	4	5	6	7	8	9
1	Involving traditional clinical professionals (e.g., surgeons, oncologists, radiation therapists, palliative care specialists, …)									
2	Involving non-traditional clinical professionals (physiotherapists, psychologists, …)									
3	Involving clinicians from partner institutions according to a hub/spokes model									
4	Involving the patient									
5	Involving the patient’s family									
6	Involving the patients’ associations									
7	Involving other NGOs (non-governmental organizations) and no-profit entities									
8	Involving other public sector entities									
9	Involving ethical committees									
10	Involving clinicians from international institutions									
11	Involving scientific societies									
12	Involving other pancreas units									
13	Involving pure research institutions and universities									
14	Involving private firms/companies active in the pharma sector									
15	Involving private firms/companies active in the biotech sector									
16	Involving private firms/companies active in the surgical technology sector									
17	Involving private firms/companies active in the media sector									
18	Involving colleagues characterized by diverse features									

#### Section 3: non-technical skills (1 + 16 items)

Non-technical skills appear today as a key topic in surgical practice. Non-technical or soft skills represent those personal features and traits that facilitate the relationships with others—namely, team members, colleagues from other disciplines, the patient and families, and other stakeholders like other institutions, communities, scientific societies. The aim of the section is to gather expert consensus on the importance of some non-technical skills. Although all of them may seem perceived as relevant, please assess them by ranking seven and up only those features that, to you, can facilitate the relationships within all the stakeholders of the pancreas unit.

**Table:**

In a pancreas unit, how would you rate the importance of non-technical or soft skills from 1 to 9, where: 1 = not important at all, 9 = very important, when it comes to pancreatic cancer care?		Importance								
		**1**	**2**	3	4	5	6	7	8	9
1	Non-technical or soft skills in general terms									

**Table:**

In a pancreas unit, how would you rate the importance of the following items from 1 to 9, where: 1 = not important at all, 9 = very important, when it comes to non-technical skills in pancreatic cancer care?		Importance								
		1	2	3	4	5	6	7	8	9
1	Decision-making									
2	Coping with fatigue									
3	Communication									
4	Leadership									
5	Situational awareness									
6	Managing stress									
7	Team working									
8	Ideas creation ability									
9	Coordination ability									
10	Multicultural ability									
11	Planning ability									
12	Learning ability									
13	Professionalism									
14	Information management ability									
15	Agility									
16	Ethics									

#### Section 4: processes and knowledge translation facilitators (31 items)

The relationships among the various actors in a pancreas unit and among different pancreas units (from the surgical and the clinical staff to the patients, from the other institutions to the scientific societies) can be tough. The aim of the section is to gather expert consensus on the importance of some tools and facilitators that can enhance the outcome of such relationships. Although all of them may seem perceived as relevant, please assess them by ranking seven and up only those tools that, to you, can facilitate the relationships within all the stakeholders of the pancreas unit.

**Table:**

In a pancreas unit, how would you rate the importance of the following items from 1 to 9, where: 1 = not important at all, 9 = very important, when it comes to facilitators in pancreatic cancer care?		Importance								
		1	2	3	4	5	6	7	8	9
1	The pathogenesis and mechanisms behind diseases									
2	Mobile electronic medical records and online tools									
3	Design									
4	Web portals									
5	Image tagging									
6	Lesson learned and best practices									
7	Tours to share experiences with others									
8	Committees and meetings									
9	Journal publications									
10	In-person visit and talking									
11	Establishment of mixed teams									
12	Co-production									
13	Leaflets and brochures									
14	Training									
15	Clinical cases and critical review of complicated cases									
16	Clinical guidelines									
17	Use of interpersonal skills									
18	Discussions, debates, curiosity									
19	New technological tools									
20	Mentoring and leadership									
21	Testimonials									
22	Engaging with the patient's family									
23	Empowerment									
24	Community of practice									
25	Multidisciplinary people (e.g., degree in medicine + IT)									
26	Use of evidence-based methods									
27	Quality assessment by stakeholders									
28	Partecipation in national and international meetings									
29	Simulations									
30	Self-assessment									
31	Use of a simple language									

### Delphi questionnaire: Round 2

Definition of new macro-topics

In a pancreas unit, how would you rate the importance of the following items from 1 to 9, where:

1= not important at all,

9 = very important,

when it comes to the organizational factors in pancreatic cancer care?

**Table:**

In a pancreas unit, how would you rate the importance of the following items from 1 to 9, where: 1 = fully disagree, 5 = neutral, 9 = fully agree, when it comes to the organizational factors in pancreatic cancer care?		Importance								
		1	2	3	4	5	6	7	8	9
1	Clinical results should be shared with industry partners (technological and surgical device producers, pharma companies, …) to enhance progress in pancreatic cancer care									
2	Industry partners should be encouraged to promote and finance projects along with pancreas units									
3	Industry partners (technological and surgical device producers, pharma companies, …) may represent meaningful stakeholders in pancreatic cancer care									
4	Patients’ associations and other nonprofit entities can provide patients and institutions with meaningful support—providing patients with assistance									
5	Patients’ associations and other nonprofit entities can provide patients and institutions with meaningful support—raising funds									
6	Patients’ associations and other nonprofit entities can provide patients and institutions with meaningful support—spreading information about the care path									
7	Disseminating general information about pancreatic cancer can be helpful for the population									
8	Social media networks represent a solid channel for disseminating general information about pancreatic cancer									
9	The press represents a solid channel for disseminating general information about pancreatic cancer									
10	Clinical results should be shared among the pancreatic surgical community to enhance progress in pancreatic cancer care									
11	Clinical results should be shared among the multidisciplinary pancreatic medical community to enhance progress in pancreatic cancer care									
12	Experimental trials can enhance progress in pancreatic cancer care									
13	The availability of electronic medical records and other digital patients’ data can facilitate work within the multidisciplinary care team									
14	There are effective e-health tools that pancreatic surgeons can use to take care of their patients, e.g., for teleconsultation									
15	Clinical results should be shared with industry partners in the e-health business to enhance progress in designing and experimenting new telemedicine tools for pancreatic cancer care									
